# Sudden death in epilepsy: the overlap between cardiac and neurological factors

**DOI:** 10.1093/braincomms/fcae309

**Published:** 2024-10-01

**Authors:** Nathan A Shlobin, Roland D Thijs, David G Benditt, Katja Zeppenfeld, Josemir W Sander

**Affiliations:** Department of Neurological Surgery, Feinberg School of Medicine, Northwestern University, Chicago, IL 60611, USA; Stichting Epilepsie Instellingen Nederland (SEIN), 2103 SW Heemstede, The Netherlands; Department of Neurology and Clinical Neurophysiology, Leiden University Medical Center, 2333 ZA Leiden, The Netherlands; Stichting Epilepsie Instellingen Nederland (SEIN), 2103 SW Heemstede, The Netherlands; Department of Neurology and Clinical Neurophysiology, Leiden University Medical Center, 2333 ZA Leiden, The Netherlands; UCL Queen Square Institute of Neurology, NIHR University College London Hospitals Biomedical Research Centre, London WC1N 3BG, UK; Cardiac Arrhythmia and Syncope Center, University of Minnesota, Minneapolis, MN 55455, USA; Department of Cardiology, Leiden University Medical Centre, Albinusdreef 2, 2333 ZA Leiden, The Netherlands; Stichting Epilepsie Instellingen Nederland (SEIN), 2103 SW Heemstede, The Netherlands; UCL Queen Square Institute of Neurology, NIHR University College London Hospitals Biomedical Research Centre, London WC1N 3BG, UK; Chalfont Centre for Epilepsy, Chalfont St Peter SL9 0RJ, UK; Department of Neurology, West China Hospital, Sichuan University, Chengdu 610041, China

**Keywords:** epidemiology, seizure, sudden arrhythmic death syndrome, sudden cardiac death, sudden unexpected death in epilepsy

## Abstract

People with epilepsy are at risk of premature death, of which sudden unexpected death in epilepsy (SUDEP), sudden cardiac death (SCD) and sudden arrhythmic death syndrome (SADS) are the primary, partly overlapping, clinical scenarios. We discuss the epidemiologies, risk factors and pathophysiological mechanisms for these sudden death events. We reviewed the existing evidence on sudden death in epilepsy. Classification of sudden death depends on the presence of autopsy and expertise of the clinician determining aetiology. The definitions of SUDEP, SCD and SADS lead to substantial openings for overlap. Seizure-induced arrhythmias constitute a minority of SUDEP cases. Comorbid cardiovascular conditions are the primary determinants of increased SCD risk in chronic epilepsy. Genetic mutations overlap between the states, yet whether these are causative, associated or incidentally present is often unclear. Risk stratification for sudden death in people with epilepsy requires a multidisciplinary approach, including a review of clinical history, toxicological analysis and complete autopsy with histologic and, preferably, genetic examination. We recommend pursuing genetic testing of relatives of people with epilepsy who died suddenly, mainly if a post-mortem genetic test contained a Class IV/V (pathogenic/likely pathogenic) gene variant. Further research may allow more precise differentiation of SUDEP, SCD and SADS and the development of algorithms for risk stratification and preventative strategies.

## Teaser

Shlobin *et al*. reported that classification of sudden death in epilepsy depends on the presence of autopsy and expertise of the clinician determining aetiology. Risk stratification for relatives requires a multidisciplinary approach involving review of clinical history, toxicological analysis and complete autopsy with histologic and, preferably, genetic examination.

## Graphical Abstract

**Graphical Abstract:**
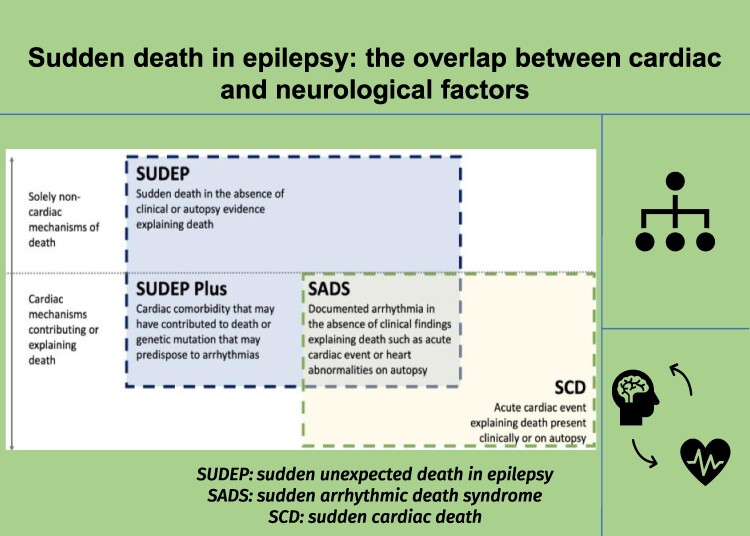

## Introduction

The term ‘sudden death’ usually describes a ‘natural’ but unexpected fatal event occurring within 1 h from the onset of symptoms in a seemingly healthy individual or in an individual for whom the disease was not deemed sufficiently severe to predict such an event. The cause is mainly cardiac in origin, with the terminal event being a fatal arrhythmia in most cases. In epidemiologic usage, sudden death refers to death occurring within 1 h of symptom onset but excluding time associated with ongoing resuscitation efforts.

Sudden death is common, may occur at any age and may have cardiac, neurologic or multifactorial aetiologies. People with epilepsy are at higher risk for premature death and have lower life expectancy than the general population.^1-3^ Sudden unexpected death in epilepsy (SUDEP) represents a common category of premature death in people with epilepsy.^4^ Other aetiologies of sudden death include sudden cardiac death (SCD) and sudden arrhythmic death syndrome (SADS) in autopsy-negative cases with negative toxicology. There is a pressing lack of knowledge regarding the risk factors, pathophysiology and prevention of all three causes of sudden death.

Often, differentiating among SUDEP, SCD and SADS is not possible in individual cases due to lack of evidence. Herein, we define and describe the spectrum of sudden death in epilepsy and distinguish SUDEP from the pathophysiology and risk factors of SCD and SADS in the epilepsy population. We discuss similarities and differences between the different types of sudden death in the epilepsy population. We also present a framework for the overall diagnostic evaluation after an episode of sudden death and highlight key research priorities to clarify the overlap between SUDEP, SCD and SADS in people with epilepsy.

## Materials and methods

A review on sudden death in epilepsy was performed using PubMed and Google Scholar from November 2022 to December 2023. Relevant articles are summarized.

### Definitions

#### SUDEP

The 2012 SUDEP definition is widely used and defines SUDEP as a sudden death not due to identifiable causes in a seemingly healthy individual with epilepsy. Various types have been defined (Table 1).^5^ This definition does not allow differentiation of SUDEP cases that occur with and without seizures and lacks clarity regarding the importance and weight of simultaneous pathological processes.^6,7^ The application of this definition has challenges. SUDEP is often used as a catch-all term. Many cases are classified as ‘possible SUDEP’ due to inadequate information.^8^ Studies often fail to list whether cases of SUDEP have definite or probable SUDEP. A study of over 1500 epilepsy-related deaths indicated disagreements between two specialists in differentiating Definite SUDEP Plus Comorbidity from Possible SUDEP and Resuscitated SUDEP.^9^ The latter is someone with epilepsy who is resuscitated and survives >1 h after cardiopulmonary arrest from SUDEP.^9^

**Table 1:** Types of SUDEP according to Nashef *et al*.^5^

Type of SUDEP	Definition
Definite SUDEP	Clinical criteria are met, and no other possible cause of death is found on anatomical and toxicological post-mortem examinations. Evidence of a terminal seizure may or may not be present, and status epilepticus must be excluded
Definite SUDEP Plus	Definite SUDEP + comorbidity other than epilepsy identified before or after death may have contributed to the death
Probable SUDEP	Clinical criteria are met, but no autopsy is available or feasible
Probable SUDEP Plus	Probable SUDEP + comorbidity that may have contributed to death
Possible SUDEP	There is evidence for a competing cause of death
Near SUDEP	A person with epilepsy who survives resuscitation for >1 h after a cardiorespiratory arrest that is not due to another identified disorder
Near SUDEP Plus	Near SUDEP + comorbidity that may have contributed to cardiorespiratory arrest
Not SUDEP	The apparent cause of death is not SUDEP
Unknown	Incomplete information

#### SCD

In 2017, the American Heart Association, American College of Cardiology and Heart Rhythm Society jointly defined SCD as ‘sudden and unexpected death occurring within an hour of the onset of symptoms, or occurring in people found dead within 24 h of being asymptomatic. Death is presumably due to cardiac arrhythmia or haemodynamic catastrophe’.^10^ This definition is not limited to arrhythmias. The 2022 European Society for Cardiology (ESC) guidelines, endorsed by the Association for European Paediatric and Congenital Cardiology, provided a more precise definition.^11^ SCD is ‘sudden natural death presumed to be of cardiac cause occurring within 1 h of onset of symptoms in witnessed cases and within 24 h of last being seen alive when unwitnessed.’^11^ Both definitions are challenging to implement because the timeline often cannot be elucidated in many instances, and non-cardiac causes may be included. Time employed during active resuscitative measures is not included.

#### SADS

The concept of SADS has evolved. Definitions have been proposed primarily by single studies with varying inclusion criteria. The 2022 ESC guidelines provided a formal definition for SADS.^11^ It is defined as sudden unexplained death occurring within 1 h of the onset of symptoms in witnessed cases or within 24 h of last being seen alive in unwitnessed instances in an individual over 1-year-old with negative autopsy (including brain autopsy), pathological and toxicological assessment. SADS is synonymous with autopsy-negative sudden unexplained death in individuals >1 year of age. In contrast, sudden infant death syndrome is a death meeting these same criteria in someone younger than 1 year old.^11^ We utilize the 2022 ESC definition of SADS as our definition.

### Epidemiology and risk factors

#### SUDEP

The apparent risk of SUDEP varies by the definition used, the nature of the population studied and the study methodology. The incidence is lowest in unselected population-based cohorts at 0.09–0.35 per 1000 person-years.^8^ Incidence rates per 100 000 person-years range from 0.9–2.3 in general epilepsy populations and 1.1–5.9 in people with drug-resistant epilepsy. SUDEP is the leading cause of premature death in epilepsy among children and adults, accounting for 10–50% of deaths in people with chronic epilepsy.^8^ In a prospective cohort of individuals diagnosed with epilepsy in childhood with a median 40-year follow-up, SUDEP accounted for about a third of all deaths, almost all in adulthood.^1^

Symptomatic epilepsy and increased seizure frequency are associated with SUDEP.^12^ Convulsive (focal to bilateral or generalized tonic-clonic) seizures are associated with a nearly 30-fold increase in risk.^12^ The presence of nocturnal convulsive seizures further increases SUDEP risk,^12-14^ while enhancing nocturnal supervision (e.g. through sharing a bedroom or using a listening device) reduces SUDEP risk.^12,13,15^ An odds ratio for SUDEP of 67 was reported in people having convulsions and not sharing bedrooms.^12^ These findings suggest that witnesses could terminate a potential SUDEP event by ending seizures, repositioning, oropharyngeal suctioning or administering oxygen.^16^ Providing direct proof for this assumption is challenging, and how often witnesses intervene is unknown. Sharing a bedroom may also be protective by allowing cohabitants to reduce the frequency of seizures through optimal medication management or individual education.^17^

The risk of definite or probable SUDEP is >7-fold lower for individuals receiving anti-seizure medications (ASMs) at efficacious doses relative to individuals receiving placebo,^18^ highlighting the importance of effective anti-epilepsy treatment. Reports of non-adherence in the medical record were associated with an odds ratio of nearly 3 for SUDEP risk.^19^ The association of individual ASMs with SUDEP risk is unclear.^19^ ASM polytherapy has been associated with reduced SUDEP risk.^19^ There may be, however, no difference in risk associated with monotherapy, polytherapy or particular ASMs when controlling for the number of convulsive seizures or other confounding variables. Epilepsy surgery and vagus nerve stimulation are associated with decreased SUDEP risk.^20^

Statin use has also been associated with reduced SUDEP risk,^19^ but it is unclear whether this observation is a proxy for general medication adherence or indicates decreased cardiovascular risk. Substance use disorders or alcohol dependence have been associated with SUDEP.^12^ Male sex and comorbid learning disabilities may be linked to SUDEP, but the evidence is limited.^12^ Other less frequently reported risk factors include history of a significant neurologic insult,^21^ low Apgar score and small for gestational age.^22^

#### SCD

Existing estimates of SCD epidemiology have limitations, including varied definitions, heterogeneous data sources and case ascertainment strategies, and constraints in excluding non-cardiac causes of death.^23^ The incidence of SCD ranges from 14.9 to 110.8 per 100 000 person-years in unselected cohorts and varies by region.^23^ Approximately, 340 000 cases of SCD occur annually in the European Union.^24^ The numbers are similar in the USA.^25,26^ Studies focused on adults over 18 report a higher incidence than those addressing younger age groups.^23^ A Chinese study found that although the mean age at SCD was lower for men (66.7 ± 16.9 years for men and 73.8 ± 13.5 years for women), there was no difference in incidence between men and women (44.6 per 100 000 per year for men and 39.0 per 100 000 annually for women).^27^ Reported ‘SCD’ often occurs ≥2 h after the onset of warning symptoms,^28^ though this may not be true SCD per the modern definitions.

Ischaemic heart disease is the most common cause of SCD in the western world. However, a wide range of other conditions has been associated with SCD, including non-ischaemic heart disease (many of the latter are believed to be genetically determined), hypertension, diabetes, dyslipidaemia, cigarette smoking, obesity, atrial fibrillation, chronic kidney disease, depression, anxiety and unhealthy lifestyle factors.^23,29^ The causes of SCD are often age dependent, most frequently coronary artery disease in adults and electrical disturbances of cardiomyopathies in children.^23^ Death at night is more common among unexplained as opposed to explained SCD.^30^

Several clinical markers have been advocated as helpful for predicting increased SCD susceptibility, but they have yet to prove to be without problems. For instance, ECG abnormalities, such as high resting heart rate, markers of abnormal depolarization and repolarization, and left ventricular hypertrophy, are associated with increased SCD risk. Other more technical markers, such as abnormal signal-averaged ECG, low heart rate variability and microvolt T-wave alteration, have been advocated at various times but have not proved to be effective tools. The Oregon SCD study indicated that left ventricular hypertrophy, higher heart rate, longer QTc, longer QRS duration, delayed QRS transition zone, wider QRS-T angle or more extended T-wave peak to T-wave end predicted SCD in univariable models.^31^ An ECG risk score was developed, in which each of these abnormalities was assigned a point, and the number of abnormalities was summed.^31^ In a multivariable model, the odds ratio of SCD increased as the ECG risk score increased, and the ECG risk score ≥4 had an odds ratio of 21.2 for SCD.^31^ An observational cohort study indicated that obstructive sleep apnoea is associated with a composite outcome of myocardial infarction, coronary artery revascularization procedures or death from cardiac causes with a hazard ratio (HR) of 2.06 after adjustment for cardiovascular risk factors, probably by lowering the threshold for arrhythmias.^32^

#### SADS

SADS most often occurs in individuals aged 30–50 years of age.^33,34^ SADS accounts for approximately one-third of sudden death in people <35 years old, amounting to the most common or second most common clinical scenario for SCD, depending on the country.^35,36^ A study of individuals aged 1–35 found no gender-based difference.^37^ SADS was deemed the clinical picture for just over half of deaths during pregnancy and postpartum.^38^

In a Danish study, approximately a third of SADS victims had cardiac symptoms before death.^39^ Symptoms were present in a quarter of the population at ≥24 h before death, and one-fifth had symptoms <24 h before demise.^39^ Cardiac symptoms frequently included chest pain, dyspnoea, palpitations, pre-syncope/syncope and aborted SCD.^39^ These deaths may have been cardiac deaths, such as SCD or non-SCDs, but not within 1 h, rendering the designation as SADS inappropriate.

Comprehensive familial evaluation has identified a definite or possible/probable inherited disease in approximately half of SADS decedents, including long QT syndromes, Brugada syndrome and catecholaminergic polymorphic ventricular tachycardia.^40,41^ Early repolarization is also more common in SADS family members than controls, with an odds ratio of 5.1 (95% confidence interval 3.4–7.8).^42^ Ascending and horizontal early repolarization are more common in SADS relatives.^42^ In particular, J-point elevations, defined as J-point amplitude of ≥0.1 mV, are associated with an increased risk of SCD.^43^ J-point elevations were most commonly found in the anterior leads,^43^ though anterior early repolarization is thought to be low risk.^44^ Current evidence indicates that sudden death risk is most significant for early repolarization in the inferior or infero-lateral ECG leads. The appearance of anterior lead findings may be a coincidence rather than an arrhythmic indicator.^45^ High-risk features include J waves ≥2 mm, dynamic J-point changes and J waves with horizontal/descending ST segments.^46^

#### SCD/SADS spectrum in epilepsy

Data on SCD/SADS in people with epilepsy are scarce or may have been labelled as (probable) SUDEP. Several studies evaluated the general population’s aetiologies of post-mortem-negative sudden death. Seven per cent of those whose death was classified as SADS had a diagnosis of epilepsy, higher than the general prevalence of epilepsy of <1%,^47^ while <5% had prior syncope or documented arrhythmia.^33^ In a study of sudden death in infants and children, 86% of those with epilepsy had deaths attributed to SADS.^48^ A structurally normal heart was found in 63% of adolescents who had SCD in the UK.^49^ A notable limitation is that there is a lack of validity of an epilepsy diagnosis in SCD/SADS after death, and only those determined to have epilepsy before death should be considered reliable.

A review of hospital records of SADS cases found that one-fifth had lifetime seizure-like events; of that group, two-fifths were diagnosed with epilepsy.^39^ An extensive study of post-mortem-negative SCD victims examined 967 cases with a mean age of 31 ± 14 years, 61% male.^33^ The study reported that deaths classified as SADS are more common during sleep or at rest than during exercise.^33^ A prospective study of sudden unexpected death determined that 34% of individuals with epilepsy and a witnessed sudden cardiac arrest had evidence of seizure activity before the arrest.^50^ In total, 26% of those with epilepsy who had SCA presented with ventricular tachycardia/ventricular fibrillation relative to 44% of people without epilepsy who had SCA, and rates of survival to hospital discharge after attempted resuscitation were nearly 5-fold lower in the epilepsy cohort compared with the non-epilepsy cohort.^50^

A recent population-based UK study with a follow-up period of over 10 years reported an increased risk of overall cardiac arrhythmias (HR 1.36) and arrhythmia subtypes, including atrial fibrillation, ventricular arrhythmias and bradyarrhythmias, in people with epilepsy.^51^ The associations were not modified by genetic predisposition as indicated by polygenic risk scores. Individuals with epilepsy using ASMs, especially carbamazepine and valproic acid, had a higher risk for cardiac arrhythmias. This observation was further supported by drug target Mendelian randomization results. One study suggested that people with epilepsy have a 3-fold increased risk for sudden cardiac arrest.^52^ Sudden cardiac arrest was defined as an out-of-hospital setting cardiac arrest with ECG-documented ventricular fibrillation and differs from SCD in that SCD does not require out-of-hospital death or ECG documentation, which may be at an academic difference rather than applicable to daily care. This prospective community-based study collected cardiopulmonary resuscitation efforts for out-of-hospital cardiac arrest and stored automated external defibrillator ECG recordings.^53^ A follow-up analysis determined that cardiovascular disease, not epilepsy characteristics, is the primary determinant of ventricular fibrillation or ventricular tachycardia in people with epilepsy.^54^

ECG markers associated with SCD have been examined to study mortality risk in epilepsy. QTc ≥ 445 ms predicted all-cause mortality in people with epilepsy (HR 1.48) after adjustments for age, Charlson comorbidity index^55^ and sex in a retrospective 15-year cohort study.^56^ A Latvian study of 481 people reported an association between short QTc < 360 ms and left ventricular hypertrophy and remote death following admission because of a convulsive seizure.^57^ Table 2 lists risk factors for SUDEP, SCD and SADS in people with epilepsy.

**Table 2:** Risk factors for sudden death

	Factors associated with increased risk of SUDEP
SUDEP	Symptomatic epilepsy Convulsive seizures Increased seizure frequency Substance use disorders Male sex Comorbid learning disabilities Not sharing a bedroom Not using ASMs Not undergoing epilepsy surgery Not using statins
SCD	Heart disease Hypertension Diabetes Dyslipidaemia Cigarette smoking Obesity Atrial fibrillation Chronic kidney disease Depression Anxiety Unhealthy lifestyle factors ECG abnormalities and greater cumulative ECF abnormalities Obstructive sleep apnoea
SADS	Inherited cardiac disease

### Pathophysiology

#### SUDEP

SUDEP mechanisms remain poorly understood but have been described as a ‘perfect physiological storm’.^58^ The pathophysiology of SUDEP is probably heterogeneous. Existing case studies have used simultaneous video EEG (vEEG) and ECG to correlate SUDEP with cardiorespiratory abnormalities,^59,60^ introducing selection bias. The most common sequence of events appears to be seizure-induced electrocerebral shutdown, apnoea and asystole.^61^ A less common variant is convulsive seizure-induced ventricular arrhythmia, mainly tachyarrhythmias.^61^ Lastly, non-seizure SUDEP has been proposed as a separate entity but is likely rare and controversial. Genetic underpinnings and neurotransmitter dysfunction are yet understudied and considered as modulatory factors.^61^

##### Seizure-related cardiorespiratory changes

SUDEP is a spectrum of heterogeneous causes mainly attributable to postictal apnoea-asystole triggered by convulsive seizures.^61^ Convulsions directly impact cardiorespiratory function. Over 80% of seizures trigger tachycardia; the proportions are similar between generalized and focal convulsive seizures, most prominently in mesial temporal lobe epilepsy.^62^ Ictal asystole occurs in 0.32% of people undergoing vEEG monitoring and strongly links to temporal lobe seizures,^59^ though this has not directly been linked to SUDEP. Four studies using implantable loop recorders determined that ≥40% of individuals with epilepsy had arrhythmias auto-detected by the device. The rate of serious bradyarrhythmias was, however, overall low despite the long-term follow-up, with only 0–3% of individuals considered candidates for pacemaker insertion.^63-66^

Approximately, one-third of seizures in people with focal epilepsy are accompanied by oxygen desaturations below 90%, including one-tenth with desaturations below 80%.^67^ Ictal central apnoea is the most common phenomenon and is only seen in focal predominantly temporal lobe epilepsies.^67,68^ Seizure spread to the amygdala is associated with the onset of ictal apnoea.^69^ Postictal convulsive central apnoea is less common and seen in focal and generalized epilepsy. The occurrence of post-convulsive central apnoea in two near-SUDEP and one probable SUDEP case during follow-up suggest brainstem dysfunction after convulsive seizure.^70^

The MORTEMUS study points out that electrocerebral shutdown, defined as prolonged postictal generalized EEG suppression and postictal absence of EEG activity >10 µV in amplitude, always preceded fatal cardiorespiratory dysfunction.^60^ The utility of postictal EEG suppression as a SUDEP biomarker is debatable. A case–control study determined that postictal generalized EEG suppression of >50 s was associated with a higher risk of SUDEP.^71^ Still, this case–control study has limitations: a small sample size, overrepresentation of a few individuals and the lack of respiratory parameters.^71^ A comparable finding has not been replicated in subsequent studies.^72^

A second, less common and less well-documented cause of SUDEP includes postictal ventricular tachycardia and ventricular fibrillation following convulsive seizures.^61^ The occurrence of arrhythmias in the absence of evident cardiac pathologies suggests seizures may induce changes in cardiac properties or trigger previously unrecognized genetic or drug-induced susceptibilities.^4,51^ People with drug-resistant epilepsy often have cardiac repolarization abnormalities. Ictal QTc prolongation is seen in about 16% of seizures, possibly increasing the propensity for polymorphic ventricular tachycardia/ventricular fibrillation.^73^ Ictal asystole is unlikely to cause SUDEP as heart rate changes resemble benign vasovagal syncope and are often self-limited. Individuals with ictal bradycardia and ictal asystole have died from SUDEP despite having functioning pacemakers.^61^ This thus contrasts with postictal central apnoea, which has been documented in near-SUDEP and probable SUDEP cases and may be considered a biomarker for SUDEP.^70^

Several factors that modulate susceptibility to peri-ictal respiratory dysfunction and thereby impact SUDEP risk have been assessed. For example, respiratory dysfunction may induce cardiac repolarization abnormalities with marked individual differences in cardiac responses to hypoxaemia.^74^ Adenosine receptor activation in the brainstem may promote respiratory depression, resulting in SUDEP.^75^ A higher incidence of SUDEP in adenosine kinase knockdown mice indicates a role of A_2A_ receptor signalling in promoting SUDEP, presumably by suppressing brainstem GABAergic inhibitory neurons and their ensuing cardiorespiratory effects.^76^ The effect of adenosine may be location dependent. Individuals at increased risk for SUDEP have lower astroglial adenosine A_2A_ receptors in the temporal lobe, which might impair adenosine modulation following seizures.^77^ Postictal dysfunction of 5-HT neurons could also contribute to SUDEP by inducing depression of breathing and arousal.^78^ Interictal serotonin level is inversely associated with postictal generalized EEG suppression duration.^79^ Similarly, seizure-related increases in serum 5-HT levels are associated with a decreased incidence of seizure-related breathing dysfunction.^80^ Seizures in people taking serotonin reuptake inhibitors are associated with a lower relative risk of ictal central apnoea.^81^ It is still questionable if these findings are actionable as a nationwide recent case–control study in Sweden examining the link between medication use and SUDEP showed that selective serotonin reuptake inhibitor (SSRI) usage did not impact SUDEP rates.^19^ However, a limited number of individuals were exposed to SSRIs in this study.

##### Interictal cardiorespiratory changes

The concept of the ‘epileptic heart’ has been developed to describe the cardiac impact of chronic epilepsy.^82,83^ The epileptic heart is defined as ‘a heart and coronary vasculature damaged by chronic epilepsy as a result of repeated surges in catecholamines and hypoxaemia leading to electrical and mechanical dysfunction’.^82,83^ This may be supported by the higher rate of cardiovascular comorbidities, such as heart failure and valvular disease, in people with epilepsy.^84^ Cardiac injury may occur as a consequence of excessive calcium loading. Still, data are lacking and observed changes are not thought to result from pre-existing cardiac disease but from chronic epilepsy.^12^

Chronic epilepsy predisposes to repeated hypoxaemia, myocardial ischaemia, cardiotoxic effects of excess catecholamines^82,83^ and increased inflammatory and metabolic factors.^61^ The structural changes resulting from myocardial damage may interfere with heart excitation, conduction or repolarization,^61^ with potential modulation of the ictal autonomic response.^82,83^ Measures of ventricular function on echocardiography, including myocardial strain and left systolic or diastolic function, are impaired in people with chronic epilepsy without previous cardiovascular disease.^85,86^ Echocardiographic measurements are not associated with seizure frequency, epilepsy duration or epilepsy aetiology.^86^

The role of chronic microstructural cardiac changes in SUDEP has been scrutinized. A pathological analysis found that SUDEP cases had less gross and histologic evidence of cardiac pathology relative to SADS cases and similar cardiac pathology to epilepsy controls and individuals who had died from trauma.^87^ A meta-analysis indicated no difference in cardiac hypertrophy, heart weight or cardiac fibrosis between SUDEP cases and people with epilepsy.^88^

Coronary artery disease is more prevalent in people with epilepsy than those without in nationwide cohort studies.^84,89^ A diagnosis of epilepsy is associated with a higher number of cardiovascular risk factors and adverse cardiovascular events.^84^ Epilepsy and the use of ASMs appear to contribute to the increased prevalence of atherosclerosis, which may explain why SCD is more common in people with epilepsy.^4,90^ In particular, enzyme-inducing ASMs predispose people to dyslipidaemia, carotid intima-media thickening and hyperhomocysteinaemia.^91-93^ The relationship between coronary atherosclerosis and SUDEP requires further work.

Non-seizure-related SUDEP is rare,^94^ but people with epilepsy may have autonomic dysfunction promoting arrhythmias or apnoeas, irrespective of seizure status.^61^ A cross-sectional study determined that ECG risk markers for SCD, including early repolarization and severe QTc prolongation are more frequent among people with epilepsy compared with healthy controls.^95^ Some investigators have associated reduced short-term heart rate variability, another SCD risk marker, with SUDEP, but the utility of heart rate variability in determining SUDEP risk is unclear.^96^

Non-cardiac mechanisms may exhibit heterogeneity. Factors contributing to ictal pulmonary dysfunction include sleep-related breathing changes and positioning.^8^ Obstructive respiratory mechanisms have been suggested, including airway obstruction via ictal laryngospasm.^97-99^ Obstructive sleep apnoea occurs in up to almost half of the people with epilepsy,^100^ but it is yet unknown whether it could increase SUDEP risk. People who die as a result of SUDEP are often found in the prone position.^60^ In addition to lying prone, circadian rhythms affecting arousal and breathing, a lack of witnesses and the sleep-wake state may be responsible.^74^

##### Relationship to sudden unexpected death in childhood

Sudden unexpected death in childhood (SUDC) is defined as the unexpected death of a child older than 1 year that is unexplained after a review of the clinical history, circumstances of death and complete autopsy.^101^ Children who die from SUDC are more likely to have a personal and/or family history of seizures, often associated with fever, than healthy controls.^102,103^ Convulsion-associated SUDC cases have been witnessed, though like SUDEP, SUDC cases most commonly occur during sleep, are unwitnessed and are found in the prone position.^102-104^ Autopsy results often show minor pathological findings.^105^ Hippocampal anomalies related to those found in people with temporal lobe epilepsy are often present, including those affecting the dentate gyrus.^103,106-108^ Pathophysiological mechanisms have not been elucidated but may include brainstem autonomic and serotonergic abnormalities.^105^ It is, therefore, possible that SUDC partly overlaps with SUDEP.

### SCD/SADS

#### Primary mechanisms

SCD is most often due to a lethal arrhythmia, usually as a consequence of underlying heart disease. Various aetiologies involving numerous pathological mechanisms may contribute. Ischaemic heart disease is the most common, but forms of heart disease (e.g. non-ischaemic cardiomyopathy, valvular, genetic/familial) also contribute. Quantitating individual risk has, however, proved challenging even after identifying the potential for an underlying disease to trigger an SCD event. SADS is, by definition, not due to structural heart disease and is instead secondary to an underlying ion channel abnormality, a so-called channelopathy, as discussed later. SADS victims are likely to have primary heart disease, while SCD victims, mainly if >30–40 years old, are more likely to have coronary artery disease.^109^

Coronary artery disease is present in 20–80% of all SCDs.^110^ Among people ≥50 years old, as many as four-fifths of cases involve coronary artery disease/ischaemic cardiomyopathy and one-fifth involve dilated cardiomyopathy.^109^ As an age group, the aetiology in people <50 years is often inherited and non-ischaemic diseases, such as hypertrophic cardiopathy in as many as one-third and arrhythmogenic right ventricular dysplasia in one-quarter, with smaller proportions involving myocarditis, coronary artery disease, other coronary anomalies, valvular heart disease, dilated cardiomyopathy and channelopathies.^109^ However, in the fourth decade, at least 50% of SCDs are due to CAD, particularly acute coronary syndrome.^109^

Approximately, half of SCD is associated with ventricular arrhythmias such as ventricular tachycardia or ventricular fibrillation.^111^ In contrast, bradyarrhythmias and electromechanical dissociation tended to be related to non-cardiac death and non-SCD.^112^ However, the timing of rhythm recording impacts the percentages. Thus, early recording may document ventricular tachycardia or ventricular fibrillation. The rhythm often degenerates if the rescuers arrive much later, and asystole or electromechanical dissociation is observed. Resuscitation at that stage is generally highly unlikely, although recent advances in neuroprotective CPR have been encouraging.

Approximately, one-tenth of cases of SCD involve no underlying heart disease on autopsy and result from channelopathies or other previously undetected genetic abnormalities.^109^ These cases may be SADS given the unexplained nature of the death if there is a negative autopsy, pathological and toxicological assessment. Brugada syndrome is reported to be responsible for one-fifth of SCD in people with structurally normal hearts.^113^ Long QT syndrome, catecholaminergic polymorphic ventricular tachycardia and rarely short QT syndrome are also detected in subsets of such individuals.^113^

Physical and electrical mechanisms are interrelated in determining SCD susceptibility. Acute coronary ischaemia leads to ventricular fibrillation, while chronic structural heart disease often leads to scar-related and often monomorphic ventricular tachycardia.^114^ Channelopathies such as long QT syndrome are implicated in polymorphic ventricular tachycardia, torsades des pointes, bidirectional ventricular tachycardia or ventricular fibrillation.^114^ These rules are not solid, and as noted, an early recording of ischaemia-induced cardiac arrest will often show ventricular tachycardia, albeit nearly always polymorphic.

Myocardial scarring from a previous infarction, primarily due to coronary artery disease, is the most prevalent ventricular tachycardia substrate. Depending on the cycle length of ventricular tachycardia, cardiac function and residual ischaemia, ventricular tachycardia may degenerate into ventricular fibrillation.^114^ The pathophysiology of SCD may be conceptualized as an interaction between a transient event or trigger and an underlying substrate that produces electrical instability and ventricular arrhythmia, promoting haemodynamic collapse.^115^ Substrates include fibrosis and scarring, while ischaemia, electrolyte imbalances, increased sympathetic tone and fever are reversible triggers.^115^ These triggers may be essential in channelopathies, perhaps even gene mutation specific.

#### Other mechanisms

Acute metabolic alterations and oxidative stress, often during myocardial ischaemia, may lead to arrhythmias and SCD.^116^ Metabolic alterations and oxidative stress affect cardiac repolarization and intra-cardiac conduction. Chronic conditions may promote structural, adverse remodelling and the creation of arrhythmogenic substrates.^117^ Acute changes may exacerbate regular changes.^118^ Others have conceptualized the role of oxidative stress as a sequential process. Myocardial stress due to ischaemia and reperfusion, heart failure or diabetes tips the delicate balance between antioxidant defence systems and reactive oxygen species in favour of reactive oxygen species.^119^ Sympathetic nervous system activation and catecholamine release during a stress response may underlie many conditions leading to SCD.^120^ Catecholamine release may stimulate beta-adrenoreceptors that lead to calcium release, whereas activation of alpha-receptors may promote coronary spasm and myocardial ischaemia, platelet activation and auto-oxidation.^121-124^

Two circadian mechanisms may play a role in SCD susceptibility. First, the suprachiasmatic nucleus may directly promote arrhythmogenesis by activating the autonomic nervous system and neurohumoral factors.^125^ Second, a local circadian clock in the heart may drive cardiac ion channel expression, promoting arrhythmias.^125^ Severity of nocturnal hypoxaemia, a core feature of obstructive sleep apnoea, predicts SCD.^126^ SADS due to primary electrical disease has gene-dependent triggers, including sympathetic tone.

### Genetic underpinnings

Selected mutations implicated in or suggested to be responsible for SUDEP, SCD and SADS are shown in Table 3. It is essential to provide the caveat that Class IV (likely pathogenic) or V (pathogenic) variants are the most important. In contrast, variants of unknown significance may obscure the relationship of genetics to sudden death. General population surveys that perform genetic autopsies in people with sudden death are most informative as they provide no assumption about the cause of sudden death. In a study of the Sudden Death in the Young Registry, 3.3% had a pathogenic or likely pathogenic variant in a cardiomyopathy and arrhythmia gene panel, 1.4% in an epilepsy gene panel and 0.5% in both.^127^ The genomes of Sudden Death in the Young cases were enriched for rare, damaging variants in genes related to these conditions.^127^ Additional studies have investigated genes associated with SUDEP and SCD/SADS, as summarized in the paragraphs below.

**Table 3:** Examples of candidate genes for conditions

Condition	Candidate genes
SUDEP	*KCNA1*, *SCN1A*, *HCN2*, *PRRT* *KCNQ1*, *KCNH2*, *SCN5A*, *RYR2* *KCNT1*, *SCN5A*, HCN family, chromosome 15
SCD	Long QT syndrome: *KCNQ1*, *KCNH2*, *SCN5A* Short QT syndrome: *KCNH2*, *KCNQ1*, *SCLA4A*, *KCNJ2*, *CACNA1C*, *CACNB2* Brugada syndrome: *SCN5A* Catecholaminergic polymorphic ventricular tachycardia: *RYR2*, *CASQ2* Idiopathic ventricular fibrillation: *DPP6*, *KCNJ8*, *CACNA1C*, *CACNB2B*, *CACNA2D1*, *SCN5A*, *ACTN2* Cardiac conduction disease: *SCN5A*, *SCN5B*, *TRPM4*, *TNNI3K* Hypertrophic cardiomyopathy: *MYBPC3*, *MYH7* Dilated cardiomyopathy: *TTN*, *LMNA*, *MYH7*, *TNNT2*, *FLNC*, *PLN*, *DSP* Arrhythmogenic right ventricular dysplasia (arrhythmogenic cardiomyopathy): *PKP2*, *DSP*, *JUP*, *DSG2*, *DSC2*
SADS	*RYR2*, *SCN5A*, *HCM*, *HCM1*, *HCM3*, *LQT1 (KCNQ1)*, *LQT2 (KCNH2)*

#### SUDEP

A study using exome sequencing and variant collapsing analysis found that nearly half of SUDEP cases had an identified mutation or pathogenic variant.^128^ A mutation in a SUDEP victim does, however, not necessarily imply that the gene contributed to the fatal event. The mutation may instead be a marker of an epilepsy aetiology.^129^ The ultimate proof of an association between a gene and SUDEP is documentation that the cause of death in a person with epilepsy can be plausibly attributed to an ion channel or other mutation. This may be accomplished by recording pathophysiological changes preceding SUDEP known to correspond to the mutation; this is a challenge in clinical practice.

#### SCD/SADS

In a prospective study of people aged 1–35, a clinical diagnosis of inherited cardiovascular disease was made in over 10% of families with unexplained SCD.^30^ A clinically relevant gene mutation was identified in nearly one-third of cases of unexplained SCD in which genetic analysis was performed.^30^ Molecular autopsies found clinically actionable pathogenic or likely pathogenic variants in over 10% of SADS cases, most commonly long QT syndrome and catecholaminergic polymorphic ventricular tachycardia.^130^ Targeted exome sequencing has indicated that over one-tenth of first-degree relatives of SADS, victims carry rare or novel non-sense candidate mutations and a fifth have previously published candidate mutations for inherited cardiac conditions.^131^

Genes involved in primary electrical disorders, most commonly long QT syndrome,^132-135^ short QT syndrome,^136-140^ Brugada syndrome,^141^ catecholaminergic polymorphic ventricular tachycardia^142-144^ and less commonly cardiac conduction disease,^141,145-148^ have been implicated in SCD/SADS. Genes implicated in hypertrophic cardiomyopathy,^136,149,150^ dilated cardiomyopathy^135,136,151^ and arrhythmogenic right ventricular cardiomyopathy^152^ have been associated with SCD. Given that SCD often occurs due to coronary artery disease, genes contributing to the acceleration of coronary artery disease may also contribute, with strong environmental influence.^136^

### Overlap between SUDEP and SCD/SADS

The most common pathogenic/likely pathogenic variants identified in people with SUDEP are in genes associated with ion channels or arrhythmias, occurring in ∼11% of cases.^153^ This percentage may grow as more genetic-related channelopathies are discovered. A community-based prospective cohort study using whole-exome sequencing reported that rare variants in genes related to heart disease were found in incident SUDEP cases but not in living individuals with or without epilepsy.^154^ A smaller study using whole-exome sequencing of resected brain tissue determined that rare variants in genes associated with cardiac arrhythmia were found in SUDEP victims but not living individuals with epilepsy.^155^ These studies inform the linkage between SUDEP and SCD/SADS and reinforce the need for studies focused on elucidating the pathophysiological mechanisms of SUDEP. Other studies have attempted to identify genes associated with seizures to inform the understanding of craniocerebral channelopathies. A registry study of people with long QT syndrome indicated that there was a higher prevalence of LQTS2, KCNH2-pore and KCNH2-cNBD predicted seizures.^156^ The definition of seizure (individual/physician reports without external validation) was imprecise, such that seizures could instead represent arrhythmic syncope, rendering the association of long QT variants and epilepsy risk unclear. Further studies investigating the genes potentially involved in craniocerebral channelopathies are warranted.

In contrast, genetic structural epilepsies may pertain to SUDEP but not SCD/SADS based on limited evidence. People with genetic structural epilepsies, including tuberous sclerosis complex, may die suddenly.^157^ Recent studies have linked mutations in DEPDC5 to SUDEP,^128,158^ yet these cases do not appear to involve cardiac dysfunction. Physiological and electrophysiological testing of people with pathogenic variants in *DEPDC5* and its binding partners *NPRL2* and *NPRL3*, including those who later died suddenly, showed no evidence of impaired clinical cardiac function. At the same time, an autopsy of an individual with DEPDC5 who died suddenly yielded no cardiac injury.^159^ Genetic structural epilepsies may thus reinforce some differentiation of SUDEP from SCD/SADS. Future studies must assess the pathophysiological cascades of SUDEP associated with genetic structural epilepsies in greater detail.

### Framework for sudden death in people with epilepsy

There are important caveats to the classification of sudden death, as the definitions of SCD, SADS and SUDEP contain essential failings and lead to significant openings for overlap. For SCD, in addition to cardiac causes of death, the challenge is that seizures themselves may be fatal. A total of 40% of SCD cases in people with epilepsy are better categorized as SUDEP.^160^ SADS is defined as an unknown, autopsy-negative cause that is assumed to be arrhythmic.

The definition of SUDEP is limited because a first seizure may be fatal, meaning the person may not be diagnosed with epilepsy. Convulsive seizures may lead to postictal apnoea-asystole.^61^ Bradycardia may facilitate polymorphic ventricular tachycardia/ventricular fibrillation. A proportion of SADS cases may not be classified as SUDEP but meet SUDEP criteria. The evidence on SUDEP is also imprecise as it is often used as a ‘container’ term for sudden death in people with epilepsy despite its structured definition. A normal autopsy is required to diagnose definite SUDEP.^5^ Still, many studies do not involve autopsy, failing to distinguish definite from probable SUDEP or reporting mixed cohorts. SUDEP may also subsume other categories of sudden death, including SADS, for decedents with epilepsy under the current definitions of these aetiologies.

Additional challenges include a lack of data on SADS and the potential for misdiagnosis. Apparent seizures may be misdiagnosed as epilepsy by non-experts instead of cardiac syncope,^161^ highlighting the importance of prompt cardiac and neurological assessment with ECG and EEG and the value of long-term monitoring with an implantable loop recorder.^33^ The classification of cases as SCD/probable SUDEP or SADS/definite SUDEP currently depends on reviewing prior medical records and family history, the specialist and whether an autopsy was performed (Table 4). People classified by a cardiologist and without an autopsy are likely to be denoted as SCD, while those with an autopsy confirming the absence of structural heart disease are marked as SADS. People who are classified by a neurologist and do not have an autopsy are likely labelled as probable SUDEP, while those with an autopsy not supportive of coronary artery disease are labelled as definite SUDEP.

**Table 4:** Overlap between SUDEP and cardiac death

	Cardiologist’s classification	Neurologist’s classification
No autopsy	SCD Criteria (2022 ES C guidelines) Sudden natural death presumed to be of cardiac cause 1 h from the onset of symptoms in witnessed cases or within 24 h of last being seen alive if unwitnessed	Probable SUDEP Criteria (Nashef 2012 definition) Sudden, unexpected, witness or unwitnessed, non-traumatic and non-drowning death occurring in benign circumstances in an individual with epilepsy With or without terminal seizures Excluding documented status epilepticus No other cause of death was identified on anatomical or toxicological post-mortem examination 1 h from the onset of a known terminal event
Autopsy	SADS Criteria (2022 ES C guidelines) Unexplained sudden death occurring in an individual over 1 year old No other cause of death was identified on pathological or toxicological post-mortem examination	Definite SUDEP Criteria (Nashef 2012 definition) Sudden, unexpected, witness or unwitnessed, non-traumatic and non-drowning death occurring in benign circumstances in an individual with epilepsy With or without terminal seizures Excluding documented status epilepticus No other cause of death was identified on anatomical or toxicological post-mortem examination 1 h from the onset of a known terminal event

ESC, European Society for Cardiology; SADS, sudden arrhythmic death syndrome; SCD, sudden cardiac death; SUDEP, sudden unexpected death in epilepsy.

Classifying deaths in people with a single seizure-like event causing sudden death is challenging. Most cases of SADS would not fulfil the criteria for SUDEP when an individual has a fatal first seizure. This individual cannot be diagnosed with epilepsy, and thus with SUDEP, unless post-mortem examination reveals convincing evidence for an epilepsy diagnosis. Even in those with recurring seizures, epilepsy may be undiagnosed due to a lack of medical care, especially in low- and middle-income countries and rural settings, reluctance to seek or accept the diagnosis and insufficient knowledge among healthcare professionals or the public.^162-164^

**Figure 1:**
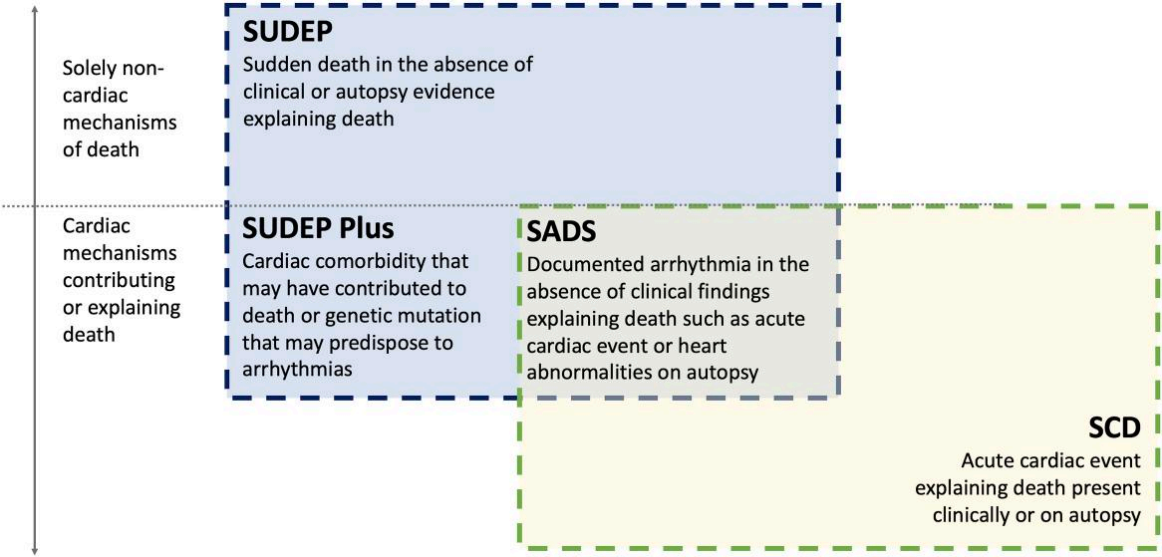
Similarities and differences in SUDEP, SADS and SCD.

We propose a framework to clarify sudden death in people with epilepsy (Fig. 1). SUDEP, SCD and SADS are heterogeneous states that overlap. Figure 1 presents the similarities and differences between SUDEP and SCD. SUDEP and SADS states require that identifiable causes of death are ruled out and that toxicology and autopsy are normal.^5,11^ An anatomically normal heart should be expected in SUDEP and SADS.^5,11^ The pathophysiological similarity between SUDEP and SADS is the occurrence of fatal ventricular fibrillation and ventricular tachycardia without an identified cause.^61,130^ Identifying these cases is a significant challenge as people with epilepsy are scarcely monitored for more extended periods by ambulatory ECGs or an implantable loop recorder. SUDEP and SCD likely include heterogeneous mechanisms.^5,115^

SADS assumes that the cause of death must be arrhythmic when toxicology, autopsy and pathology are normal.^11^ This assumption is incorrect in people with epilepsy because these examinations may not have identified causes other than arrhythmia. An example is the documentation of convulsive seizures with resultant postictal EEG suppression and apnoea leading to asystole. While strictly speaking, the SCD definition may apply, we prefer the label of SUDEP as the arrhythmia results from seizure-induced brain stem depression. Accordingly, the typical situation in which someone is found dead in bed with evidence of a recent seizure is preferably classified according to the SUDEP framework. Witnessed cases of death following a seizure may also be classified according to SUDEP.

Determining death mechanisms in cases of sudden death in people with epilepsy is vital. Improving the phenotyping of cases, including through ECGs, echocardiograms and adding cardiology panels when ordering genetic tests, may enhance our understanding of the contribution of various pathomechanisms and risk factors to sudden death in people with epilepsy. Possibly, the advent of seizure detection devices or smart wearable monitors may also help to improve the clinical evaluation of sudden death in epilepsy. Studies should carefully differentiate probable SUDEP from definite SUDEP cases to determine the relationship of SUDEP to SADS. Extensive population-based studies should identify the prevalence of epilepsy in people who die from SCD and SADS in diverse populations globally and characterize unmodifiable and modifiable risk factors for SUDEP, SCD and SADS. Identifying risk factors for SADS in distinct populations of people with epilepsy is important. Studies employing long-term cardiac monitoring with tandem seizure recording methods will help determine the role of cardiac changes and seizures in SUDEP.

SUDEP, SCD and SADS may share a common genetic basis, given that channelopathies associated with them occur in channels present in the brain and heart given the partly overlapping candidate contributory genes (Table 5).^30,165-167^ Existing genetic analyses fail yet to provide convincing evidence of the role of specific gene mutations in the pathophysiology of SUDEP, SCD and SADS. Many mutations related to any of these conditions are variants of unknown significance. It is unclear whether gene mutations are directly responsible for SUDEP, SADS or both or whether they are present in individuals who die from one or both of these states. Selection bias may lead to an illusory relationship between the mutation and SUDEP, SCD or SADS. Linking gene mutations to a causative role in the pathophysiology of each condition by establishing the temporal relationship between the dysfunction of the proteins that these genes code for and sudden death will clarify the relationship.

**Table 5:** Genetic overlap

Sudden death	Genes	Location/function
SUDEP, SCD, SADS	*RYR2*, *KCNQ1*, *KCNH2*, *SCN5A*	Ion channels expressed in the heart and brain
SUDEP and SCD	*HCN4*, *KCNE1*	Ion channel proteins
	*DPS*, *DSG2*	Cardiac desmosomes and brain
Epilepsy and SCD	*CHD2*	Chromatin remodelling
	*CHRNA4*	Nicotinic acetylcholine receptor
	*SCN8A*	Sodium channels expressed in heart and brain
	*SPTAN1*	Alpha-spectrin found in cardiac muscle and brain

SADS, sudden arrhythmic death syndrome; (SCD, sudden cardiac death; SUDEP, sudden unexpected death in epilepsy (SUDEP).

### Classification of sudden death and risk stratification in high-risk decedents

**Figure 2:**
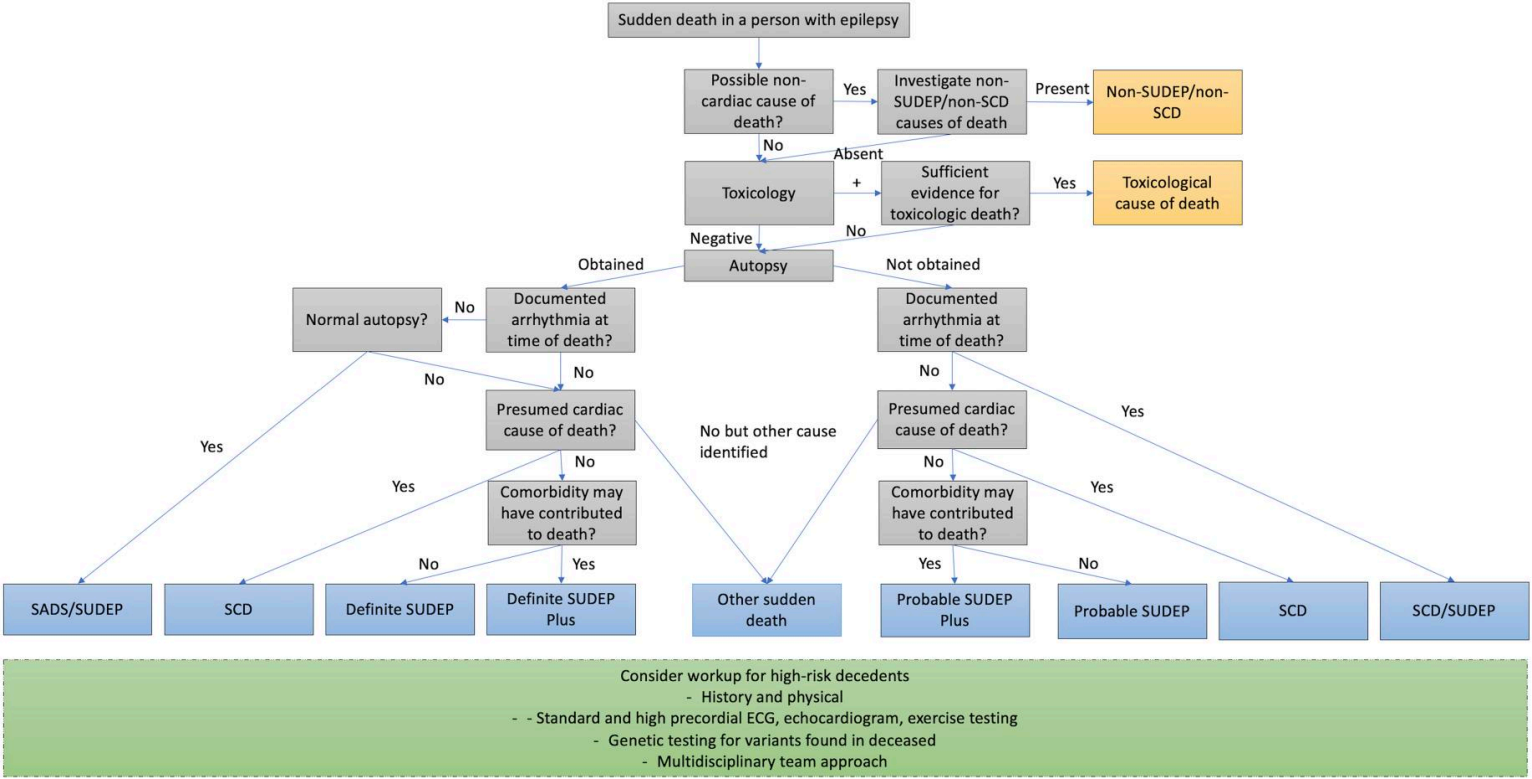
Flow chart for the work-up of sudden death in people with epilepsy.

We present a protocol to classify sudden death in people with epilepsy better and stratify risk for sudden death in high-risk relatives, namely those whose relation with sudden death had a history, physical examination, autopsy or genetic findings concerning a potentially heritable condition, based on the best available evidence (Fig. 2). Individuals who present with unexpected sudden death should undergo a review of their clinical history, toxicological analysis and complete anatomic autopsy with additional histologic analysis.^168^ During autopsy, sampling for genetic analysis should be acquired for current use and storage if later analyses are required.^168^ Post-mortem genetic testing targeted to primary electrical disease is recommended when the relative is ≤50 and the circumstances and family history support a primary electrical disease.^11^ Genetic testing should prioritize genes definitively associated with epilepsy or cardiac arrhythmias.^169^ Post-mortem genetic testing in the relative for additional genes may be considered according to clinical judgment, but hypothesis-free genetic testing using exome or genome sequencing is not recommended.^11^ Clinicians must exercise caution when attributing clinical relevance to variants in genes not definitively associated with epilepsy or cardiac arrhythmia.^169^

High-risk relatives should undergo a complete history and physical examination to identify overtly high risk.^11^ These individuals should also undergo a detailed cardiac examination with standard and high precordial ECG, echocardiogram and exercise testing.^11^ Retaining tissue from an autopsy on an individual with sudden death is essential for post-mortem genetic analysis, with yields as high as one in three.^11^ Genetic testing should be conducted in individuals with sudden death to identify pathological mutations associated with SUDEP, SCD and SADS.^11,168^ The appropriate genetic test depends on the combination of findings on anatomic and histologic autopsy, high-risk decedent history and physical examination and the results of detailed cardiac examinations.^168^ High-risk relatives should be offered genetic testing if a post-mortem genetic test in the decedent detected a Class IV (likely pathogenic) or V (pathogenic) variant.^11,168^ This process requires forming a multidisciplinary team composed of neurologists, cardiologists, geneticists and pathologists and supporting staff such as social workers and ethics and legal personnel. Additionally, this protocol inherently involves incomplete information and must be updated as further evidence arises.

## Conclusion

Sudden death in epilepsy is heterogeneous. SUDEP, SCD and SADS may be used to categorize such sudden deaths, but these conditions have overlapping heterogeneous epidemiologies, risk factors and pathophysiological mechanisms. Investigations of sudden death in epilepsy should broadly consider the heart, as SCD/SADS comprise an essential minority of SUDEP. Multidisciplinary involvement may allow stratification of sudden death risk in people with epilepsy and their relatives. Individuals with sudden death should undergo a review of their clinical history, toxicological analysis and complete autopsy with additional histologic and genetic examination. High-risk relatives of individuals with sudden death may undergo genetic testing if a pathogenic or likely pathogenic variant is identified in the deceased. Further work is needed to delineate the relationship between SUDEP and SCD/SADS to guide the development of preventative strategies.

## Data Availability

This is not applicable as this work did not generate new data. All relevant data obtained in the review are summarized in this manuscript.
